# Ceftolozane/Tazobactam Dosing Requirements Against *Pseudomonas aeruginosa* Bacteremia

**DOI:** 10.1177/1559325819885790

**Published:** 2020-01-29

**Authors:** Jesus Ruiz, Alejandra Ferrada, Miguel Salavert, Mónica Gordon, Esther Villarreal, Álvaro Castellanos-Ortega, Paula Ramirez

**Affiliations:** 1 Intensive Care Unit, IIS La Fe. Hospital Universitari i Politècnic La Fe, Valencia, Spain; 2 Pharmacy Department, Hospital Universitari i Politècnic La Fe, Valencia, Spain; 3 Infectious Disease Department, Hospital Universitari i Politècnic La Fe, Valencia, Spain; 4 Intensive Care Unit, Hospital Universitari i Politècnic La Fe, Valencia, Spain

**Keywords:** critical care, bacterial infection, sepsis, pharmacokinetics

## Abstract

**Objectives:**

To assess the probability of reaching adequate pharmacokinetic/pharmacodynamics values for ceftolozane/tazobactam at different doses and degrees of renal functions in patients with *Pseudomonas aeruginosa* bacteremia.

**Methods:**

Six dosing regimens were evaluated: 0.5/0.25 g, 1/0.5 g, and 2/1 g every 8 hours given as 1 hour or 3 hours infusions. Pharmacokinetic data were obtained from the literature. Susceptibility data to ceftolozane were collected from patients with *P aeruginosa* infection treated with ceftolozane-tazobactam. Probability of reaching a fraction of time (fT) >40% minimum inhibitory concentration (MIC) and fT >100%MIC value for ceftolozane at 3 different renal clearance values was evaluated. For tazobactam, the probability of reaching an fT >40% and >70% for 3 limit values was calculated.

**Results:**

Thirty-seven strains were included. For ceftolozane, the probability of reaching a fT >40%MIC was greater than 90% for any degree of renal function. The probability of reaching a fT >100%MIC for 1 g dose infused over 1 hour and 3 hours was 82.2% and 86.4% for a creatinine clearance (ClCr) >90 mL/min. Using a 2 g dose, the probability was greater than 90% for both infusions rates. For tazobactam, the probability of reaching a value of fT >50% of the limit concentrations was greater than 90% for a ClCr of 70 mL/min. In the case of a ClCr >90 mL/min and limit concentration values ≥ 0.25 mg/mL, only extended infusions showed a probability >90%.

**Conclusions and Relevance:**

The standard doses of ceftolozane/tazobactam achieve an adequate fT >40%MIC value. However, doses of 2 g in extended infusion is necessary to reach a value of fT >100%MIC, especially in patients with an increased renal clearance and high levels of beta-lactamases expression.

## Introduction

*Pseudomonas aeruginosa* is one of the main pathogens associated with nosocomial infections worldwide,^1,2^ being responsible for serious infections in both immunocompetent and immunosuppressed patients, presenting a mortality rate ranging between 20% and 60%.^3,4^ There are multiple mechanisms of resistance described for this bacterium, including efflux pumps, the generation of beta-lactamases, or the loss of porins.^5^ During the last few years, resistance to carbapenems has significantly increased, being more than 20% in multiple published series.^6^ Ceftolozane, a new cephalosporin, has shown to have an excellent activity against various strains of *P aeruginosa*, even in those with carbapenem resistance.^7^ Its combination with tazobactam, an inhibitor of beta-lactamases, increases its spectrum of action to strains producing beta-lactamases of class A (including the types TEM-, SHV- and CTX-M) and some of class C (AmpC).^8^ Currently, it is authorized for the treatment of severe urinary and intra-abdominal infection at doses of 1/0.5 g every 8 hours,^9^ being also used as “off-label” in the management of nosocomial pneumonia with a dose of 2/1 g every 8 hours in an infusion of 1 hour.^10^ However, to date, its dosage is uncertain in those patients with bacteremia, having postulated the need to obtain concentrations of ceftolozane above the minimum inhibitory concentration (MIC) of at least 40% of the dosage interval (T > MIC) to achieve an adequate activity.^11,12^ Notwithstanding, alterations in the volume of distribution and plasma clearance of the most severe patients, as well as the unequal distribution of the MICs in the different centers, make it necessary to reevaluate the adequacy of the dosage of this drug.

The purpose of the present project is to evaluate the probability of reaching an adequate pharmacokinetic/pharmacodynamic value (pK/pD) for different dosages of ceftolozane/tazobactam in different degrees of renal functions of the patients based on the data of the MIC obtained from patients with bacteremia due to *P aeruginosa* treated with this drug.

## Material and Methods

A simulation study was conducted based on the data obtained from strains that caused infections by *P aeruginosa* placed in a tertiary hospital treated with ceftolozane/tazobactam during the period January 2014 to January 2018. The adequacy of the dose of this drug was evaluated for 3 different degrees of renal clearance (creatinine clearance [CrCl] 35, 70, and >90 mL/min). The pharmacokinetic parameters of plasma clearance (Cl) and volume of distribution (Vd) used in the simulation are shown in Table 1, being obtained from published studies.^13,14^ In all cases, a degree of plasma protein binding to 18% and 30% was assumed for ceftolozane and tazobactam, respectively.^15^ The MIC values were calculated using the E-Test technique.

**Table 1.** Pharmacokinetics Parameters Used in the Simulation Model.

	ClCr = 35 mL/min		ClCr = 70 mL/min		ClCr >90 mL/min	
Parameter	Value	Range	Value	Range	Value	Range
Ceftolozane						
Cl (l/h)	1.7	1.1-3.3	4.3	3.2-6.2	6.3	5.93-6.66
Vd (L)	13.9	10.6-18.6	14.6	8.9-24.7	13.8	12.2-15.9
Tazobactam						
Cl (l/h)	7.6	5.4-10.2	16.6	12.4-23.0	24.5	23.4-25.6
Vd (L)	16.8	13.9-21.1	19.9	13.8-26.1	15.0	13.9-20.1

Abbreviations: Cl, total clearance; Vd, volume of distribution; ClCr, creatinine clearance.

From the data obtained, a total of 1000 Monte Carlo simulations were performed for the different doses studied and degrees of renal function through the Excel® program. Three different doses of ceftolozane/tazobactam (0.5/0.25, 1/0.5, and 2/1 g) were administered in infusions of 1 hour and 3 hours every 8 hours. The calculation of the fraction of time above the MIC was made by the following formula^16^:

$$\text{fT}>\text{MIC}=\;[\left({\text{t}}_{2}+{\text{t}}_{\text{inf}}\right)\;-{\text{t}}_{1}]\times (100/\text{τ})],$$

where fT > MIC is the proportion of time of the free drug in blood remaining above the MIC during the dosing interval. Here, t_inf_ indicates time of infusion, t_1_ value (hour) represents the time in which the serum concentrations reached the MIC value during the infusion phase, t_2_ (hour) the post-infusion time until ceftolozano/tazobactam plasma concentrations fall below the MIC value in the phase of elimination, and τ the interval of dosage.

The values of t_1_ and t_2_ were reached by the following formulas:

$${\text{t}}_{1}=\;(\text{Vd}/\text{Cl})\times \text{Ln}(1-((\text{MIC}-\text{f}{\text{C}}_{\text{min}})\times {\text{t}}_{\text{inf}}\times \text{Cl})/\text{D})$$

$${\text{t}}_{2}=\text{Ln}(\text{f}{\text{C}}_{\text{max},\text{ss}}/\text{MIC})\times \left(\text{Vd}/\text{Cl}\right)$$

where the values of fCmin and fCmax correspond to the maximum and minimum free concentrations (mg/L) reached in the dosing interval, Vd to the volume of distribution (L), Cl to the total clearance of the drug (mL/min), and D to the dose used. The values of Cmax and Cmin were obtained from the following equations;

$$\text{fCmax}=\text{fu}\times \text{D}/\text{Cl}\times {\text{t}}_{\text{inf}}\times (1-{\text{e}}^{-\text{Cl}/\text{Vd}\times {\text{t}}_{\text{inf}}})\times 1(1-{\text{e}}^{-(\text{Cl}/\text{Vd})\times \text{τ}})$$

$$\text{fCmin}=\text{fCmax}\times {\text{e}}^{-(\text{Cl}/\text{Vd})\times (\text{τ}-{\text{t}}_{\text{inf}})},$$

being D the dose used and fu fraction of drug not bound to plasma proteins. In the simulation model, a log-normal distribution was assumed for CL and Vd.

For ceftolozane, the cumulative fraction of response (CFR) for each dosage regimen considered a value of fT >40%MIC and fT >100%MIC was calculated by the following formula:

$$\text{CFR}=\Sigma^{\text{n}}\text{PTA}\times \text{fi},$$

being PTA the probability of target attainment the pK/pD value for each MIC value studied and fi the fraction of the population of microorganisms at each MIC category.

In case of tazobactam, since no specific MIC values were available, the probability of reaching an fT >40% and an fT >70% for 3 limit values: 0.05, 0.125, and 0.250 mg/L was calculated.^17^

## Results

A total of 35 strains of patients with *P aeruginosa* infection treated with ceftolozane/tazobactam were collected. The strains showed a MIC value ≤ 1 (n = 4, 11.4%), 1.5 (5, 17.1%), 2 (13, 37.1%), 3 (2, 5.7%), 4 (7, 20.0%), 8 (3, 8.6%), and 12 (1, 2.9%) mg/L. The sensitivity of the strains to other antimicrobials is shown in Table 2, with 25 (71.4%) cases resistant or intermediate sensitivity to meropenem and 26 (74.3%) cases to imipenem.

**Table 2.** Resistance Profile of the Stains Included in the Study.

	Resistant strains (%), (n = 35)
Meropenem	25 (71.4%)
Imipenem	26 (74.3%)
Levofloxacin	27 (77.1%)
Piperazilin/tazobactam	23 (65.7%)
Fosfomycin	24 (68.6%)
Colistin	2 (5.7%)
Ceftazidime	24 (68.8%)
Amikacin	10 (28.6%)

### Ceftolozane

**Figure 1.**
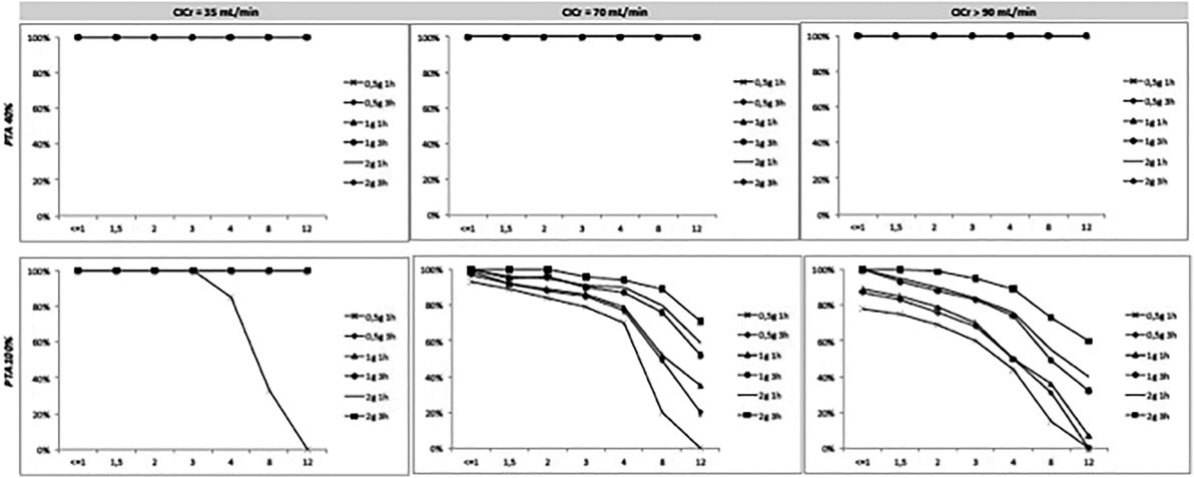
Probability of target attainment (PTA) of 40% and 100% of the dosing interval above different MICs value for different doses of ceftolozane in patients with different degrees of creatinine clearance (ClCr). MIC indicates minimum inhibitory concentration.

The PTA of ceftolozane administered in 1 and 3 hours infusions with different evaluated degrees of renal function are shown in Table 3. For any evaluated dose and degree of renal function, the probability of reaching a value of fT >40%MIC was greater than 90%. The probability of reaching the value of fT >100%MIC for the 1 g infusion dose of 1 and 3 hours was 82.2% and 86.4% for ClCr >90 mL/min and 90.3% and 94.3% for ClCr = 70 mL/ min. For the 2 g dose, the probability was greater than 90% for both infusions at 1 and 3 hours. Figure 1 shows the relation between MIC and fT >MIC for ceftolozane for the different evaluated doses and renal functions.

**Table 3.** Probability of Reaching the Parameter T >40%MIC and T >100%MIC for Ceftolozane Based on the Sensitivity of the Strains Evaluated.

T >40% MIC				
Dose	T_inf_	ClCr >90 mL/min	ClCr = 70 mL/min	ClCr = 35 mL/min
0.5	1 hour	100.0%	100.0%	100.0%
0.5	3 hours	100.0%	100.0%	100.0%
1	1 hour	100.0%	100.0%	100.0%
1	3 hours	100.0%	100.0%	100.0%
2	1 hour	100.0%	100.0%	100.0%
2	3 hours	100.0%	100.0%	100.0%
T >100% MIC				
Dose	T_inf_	ClCr = 100 mL/min	ClCr = 70 mL/min	ClCr = 35 mL/min
0.5	1 hour	62.0%	74.3%	88.6%
0.5	3 hours	67.7%	78.7%	100.0%
1	1 hour	82.2%	90.3%	100.0%
1	3 hours	86.4%	94.3%	100.0%
2	1 hour	89.7%	96.5%	100.0%
2	3 hours	95.0%	98.3%	100.0%

Abbreviations: T_inf,_ time of infusion; MIC, minimum inhibitory concentration.

### Tazobactam

**Figure 2.**
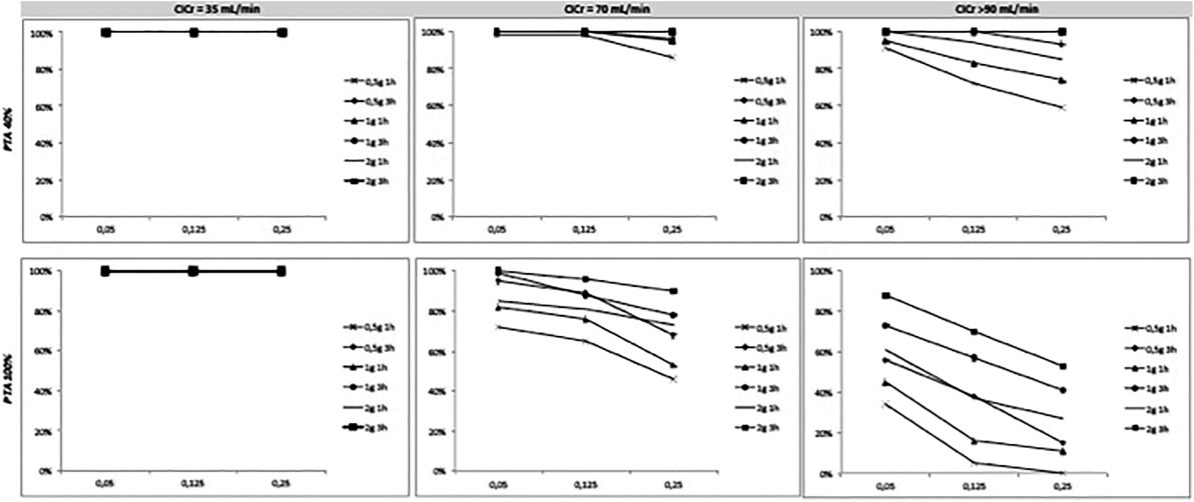
Probability of target attainment (PTA) of 40% and 100% of the dosing interval above different MICs value for different doses of tazobactam in patients with different degrees of creatinine clearance (ClCr). MIC indicates minimum inhibitory concentration.

The PTAs for tazobactam administered in the same conditions simulated for ceftolozane at different values of the evaluated limit concentrations are shown in Figure 2. For any evaluated dose and degree of renal function, the probability of reaching a value of fT >50% of the limit concentrations was greater than 90% for a CrCl of 35 and 70 mL/min. In the case of a ClCr >90 mL/min and concentration values ≥ 0.25 mg / mL, only extended infusions showed a PTA> 90%. The probability of reaching a fT >70%MIC for a Cl value = 70 mL/min was lower than 90% from a concentration value of 0.125 mg/L, increasing in case of extended infusion of 3 hours. For ClCr >90 mL/min, this probability was significantly reduced, being less than 50% for the dose of 1 g/8 hours in an infusion of 1 hour in any of the simulated limit concentration values.

## Discussion

Based on the results obtained, the recommended doses for ceftolozane are appropriate to obtain a value of fT >40%MIC in our patients. However, the objective of fT >100%MIC requires the administration of doses of 2/0.5 g of ceftolozane/tazobactam, especially in patients with a high renal clearance and with a high expression of beta-lactamases.

It is known that in serious infections, the selection of an adequate dose of antibiotics is associated with an improvement in clinical results.^18,19^ That is why the principles pK/pD have become a key element to select the dosage of antimicrobials with a higher probability of reaching the concentrations associated with a greater response to the treatment. In addition to the intrinsic resistance of the bacterial strains to antibiotics, variations in the volume of distribution, plasma clearance, or protein binding have a great influence on the concentrations reached by them in the infectious focus and therefore in the probability of response to the treatment.^20^ In our model, we have included important variations in the volume of distribution and clearance to simulate the wide variability of situations that occur in patients with bacteremia, especially in those patients in a situation of sepsis.

Ceftolozane, like the rest of beta-lactam drugs, has a time-dependent activity, conditioned to the value of fT >MIC. The initial animal models have suggested that bacteriostatic activity of this drug is sufficient when an fT >24.8%MIC is reached, reducing the bacterial load by 2 log with a fT >40%MIC.^12^ However, these same authors observed that higher concentrations achieve a higher bactericidal potential. In critically ill patients with bacteremia and severe sepsis or septic shock, the need for rapid bactericidal action is of great importance. Several authors have shown that for an adequate bactericidal action in serious patients, drugs such as carbapenems should reach a concentration of fT >100%MIC.^21,22^ Under this premise, we have simulated the ability of ceftolozane to reach concentrations values above the MIC for 40% and 100% of the dosing interval. That is why the extended infusions of beta-lactam drugs, by increasing the probability of reaching these objective parameters, have become a widely recommended practice for the management of serious infections.

In this sense, according to our simulation model, doses of 2 g in extended infusion are necessary for patients with high clarifications to achieve a PTA >90%, especially for those patients with a high clearance. It is therefore of great importance to identify this group of patients to optimize their dosages. It should be noted that in our centre, we have found higher MIC values than in previous studies,^23,24^ with 74.2% of the strains finding a value equal to or greater than 2 mg/L. This may be due to the fact that this drug has been used in our patients with strains of *P aeruginosa* with multiple resistances. This phenomenon has forced to reexamine the adequacy of the doses used of this drug.

In the case of tazobactam, since there is no intrinsic antimicrobial activity, different limit concentration values have been used in the simulation model, as in previous studies.^14^ As with other beta-lactamases inhibitors, a minimum concentration is necessary to neutralize the activity of these enzymes. For tazobactam, the inhibition of the beta-lactamases has been shown to be, like the beta-lactams, time-dependent,^17-24^ showing a dependent potential on the degree of expression of them by the bacterial strain. In our study, we used as limit concentration for tazobactam values from 0.05 to 0.25 μg/mL, the same as those used by VanScoy and colleagues^17^ in strains of *Escherichia coli* with low and high degree of expression of beta-lactamases type CTX-M-15. In this study, the exposure times above the limit concentration for tazobactam associated with a reduction of colony forming units of 1 and 2 log _10_ were from an fT >MIC of the 50% and 70%. However, as these authors argue, the limit concentration is still unknown in those strains with different resistance mechanisms, including different types of beta-lactamases, which adds a high degree of uncertainty to the necessary doses of this drug.

According to our study, for patients with a high CrCl, the concentration of tazobactam may be insufficient in those patients with a high degree of beta-lactamases expression. Even in those with a ClCr = 70 mL/min, we have obtained low plasma concentrations, therefore it is necessary to optimize the dosage of the drug, increase its dose, and/or increase the infusion time to achieve adequate exposure to it. It should be borne in mind that tazobactam has a clearance for more than 3 times higher than ceftolozane, so in those patients with high levels of enzymatic expression, its concentration could be compromised, being the limiting factor to justify a change in the dose of this drug. Cases of development of resistance in the treatment course with ceftolozane-tazobactam have been described,^25,26^ having been associated with the obtained concentrations.^27^ Therefore, an early optimization of the dosage could reduce, to a great extent, the development of resistance to it.

It should be noticed that the increased dose of ceftolozane/tazobactam could be associated with an increase in the occurrence of adverse effects. Although ceftolozane/tazobactam has shown a good safety profile at high doses in healthy volunteers,^28^ they can produce significant adverse effects such as elevation of liver enzymes, hypotension, or cutaneous reactions,^9^ although these effects have not been directly related to an increase in the doses of the drug.^28^ However, the risk benefit of the dose increase must be considered, minimizing its use to those patients in whom, due to its high drug clearance, it is really necessary.

Among the limitations of our study, the uncertainty in the values of Cl and Vd used in the model are noticed, not yet having information on these parameters in more critical patients. Recent studies have shown a great variability in ceftolozano/tazobactam Cl and Vd in critically ill patients, showing that extended or continuous infusion are needed in those patients with infection due to high MIC strains.^29^ This wide variability in the pharmacokinetic parameters invites to design future studies in specific subgroups of critically ill patients to obtain more homogeneous results that allow an improved dose optimization. In fact, information regarding the clearance of the drug in patients with a glomerular filtration rate greater than 120 mL/min is scarce. This phenomenon of hyperfiltration, frequent in critically ill patients,^30^ could significantly reduce the concentrations of both ceftolozane and tazobactam. On the other hand, we have not considered the ability of the predicted concentrations to access the infectious focus of the patient. It is known that the penetration of the drug into the lung, abscesses, or other focuses supposes a concentration lower than that reached in the blood. Ceftolozane has shown adequate penetration in lung tissue, close to 50%, with 2 g doses, being sufficient for an adequate T >MIC value of 40%.^14^ Nevertheless, as with patients with bacteremia, severe patients with a high volume of distribution or clearance should receive high doses of extended infusions to ensure adequate exposure.

## Conclusion and Relevance

The standard doses of ceftolozane/tazobactam achieve an adequate pK/pD value. Still, doses of 2 g in an extended infusion of 3 hours are necessary to reach a value of fT >100%MIC, especially in those patients with a high plasma clearance. Special caution should be considered for tazobactam patients with high clearance and high levels of beta-lactamases expression.
